# Epidemiology of *Brucella* Infection in Small Ruminants in the United Arab Emirates

**DOI:** 10.1155/tbed/6666896

**Published:** 2025-06-10

**Authors:** Gobena Ameni, Aboma Zewude, Berecha Bayissa, Ibrahim Abdalla Alfaki, Abdallah A. Albizreh, Naeema Alhosani, Meera Saeed Alkalbani, Mohamed Moustafa Abdelhalim, Assem Sobhi Abdelazim, Rafeek Aroul Koliyan, Kaltham Kayaf, Mervat Mari Al Nuaimat, Robert Barigye, Markos Tibbo, Yassir Mohammed Eltahir

**Affiliations:** ^1^Department of Veterinary Medicine, College of Agriculture and Veterinary Medicine, United Arab Emirates University, Al Ain City, Abu Dhabi Emirate, UAE; ^2^Vaccine Production and Drug Formulation Directorate, National Veterinary Institute, Debre Zeit, Oromia Region, Ethiopia; ^3^Department Business and Statistics, College of Economics and Business, United Arab Emirates University, Al Ain City, Abu Dhabi Emirate, UAE; ^4^Department of Geography, College of Humanities and Social Sciences, United Arab Emirates University, Al Ain City, Abu Dhabi Emirate, UAE; ^5^Extension Services and Animal Health Division, Abu Dhabi Agriculture and Food Safety Authority, Abu Dhabi City, Abu Dhabi Emirate, UAE; ^6^Animal Development and Health, Ministry of Climate Change and Environment, Dubai City, Dubai Emirate, UAE; ^7^Subregional Office for the Gulf-Cooperation Council States and Yemen, Food and Agriculture Organization of the United Nations, Abu Dhabi City, Abu Dhabi Emirate, UAE

**Keywords:** *brucella* infection, dromedary camels, molecular detection, seroprevalence, small ruminants, united arab emirates

## Abstract

Small ruminants are important livestock species, which function as a major source of protein, in the United Arab Emirates (UAE), although infections such as *Brucella* infection can hamper their productivity. However, there is currently a paucity of epidemiological data on *Brucella* infections in small ruminants in the UAE. This study therefore aimed to estimate the seroprevalence of *Brucella* infection and evaluate the associated risk factors in 272 flocks encompassing 2730 small ruminants in the Emirate of Abu Dhabi. In addition, DNA of the *Brucella* was tested in seropositive small ruminant. Multispecies competitive enzyme-linked immunosorbent assay (cELISA) and multispecies indirect ELISA (iELISA) were used to detect *Brucella* antibodies, while real-time quantitative polymerase chain reaction (qPCR) was used to detect the DNA of genus Brucella and its major species (*Brucella* (*B*.) *abortus*, *B. melitensis*, and *B. ovis*). Flock seroprevalences of *Brucella* infection were 13.6% (95% CI: 9.8%–18.3%) and 25.5% (95% CI: 20.3%–31.0%) based on iELISA and cELISA, respectively. While animal seroprevalences were 2.31% (95% CI: 1.8–2.9) and 4.84% (95% CI: 4.1–5.7) on the basis of iELISA and cELISA, respectively. Flock seroprevalence was associated with flock size, whereas animal seroprevalence was associated with region, holding type, species, and age. The genus *Brucella* DNA was detected in the sera of 28.21% (11/39) of seropositive small ruminants. The 11 *Brucella* genus positive sera were further identified into three *B. ovis*, three mixed infections of *B. melitensis* with either *B. abortus* or *B. ovis*, two *B. melitensis*, and one *B. abortus*. While the remaining two were not positive for any of the three species. In conclusion, although animal seroprevalences were low by both ELISA tests, flock seroprevalences were relatively high. Besides, *B. melitensis* was the dominant species that was detected in the sera small ruminants posing zoonotic threat to the public. Therefore, the results of this study warrant for re-enforcement of the control and preventive measures of *Brucella* infections in small ruminants.

## 1. Introduction

Brucellosis is a zoonotic disease caused by gram-negative coccobacillus species of the genus *Brucella* [1, 2]. Twelve species within this genus have thus far been identified in various hosts [2]. *Brucella* species exhibit a certain level of host specificity; the primary hosts of *Brucella abortus* (*B. abortus*) *B. melitensis/B. ovis*, *B. suis*, and *B. canis* are cattle, small ruminants, pigs, and dogs, respectively [3]. *B*. *melitensis* and *B*. *abortus* are widespread species associated with huge public health and economic impacts [4].

Raising small ruminants is a preferred agricultural practice in marginal environments with scarce grazing and unfavorable climatic conditions. In addition, raising small ruminants involves a rapid generation turnover, resulting in a shorter duration of pregnancy and a lower milk supply for immediate household consumption [5]. The Food and Agriculture Organization [5] report indicated that ~97% of the world's goat population is located within Asia, Africa, and Latin America, while Asia alone accounts for 51% of the world's small ruminant production. As such, the infection of small ruminants with *Brucella* in these regions could exert severe economic and public health impacts.

Brucellosis is responsible for economic losses in the agricultural sector owing to abortions, reduced milk yield, and infertility [6]. In addition, this disease can be transmitted to humans via the consumption of unpasteurized milk and dairy products, as well as direct contact with afterbirth and aborted materials. Hence, individuals with occupational contact, such as farmers, abattoir workers, shepherds, and veterinarians, are deemed to be at high risk of brucellosis [7, 8]. Regarding the magnitude of brucellosis zoonosis, a review published in 2006 indicated that brucellosis causes more than 500,000 new cases worldwide annually [9].

Small ruminants are important livestock species raised in the United Arab Emirates (UAE), with an estimated number of 5 million [10], contributing to both food security and the national economy of the UAE [10]. However, brucellosis can affect small animals and hinder their productivity. Several prior studies have been published on *Brucella* infection in small ruminants in the UAE [11–14]. However, these studies were conducted several years ago; hence, the current status of *Brucella* infection in small ruminants remains unclear. This is particularly important, as the number of small ruminants in the UAE has increased significantly over the last two decades. As such, generating epidemiological data on *Brucella* infection in small ruminants will aid in planning appropriate control and preventive measures. Of the existing research, one recently published review reported an incidence rate of 47 human brucellosis cases per 100,000 individuals [15]; this data could indirectly reflect the disease burden in animals, as human infections are acquired from animals.

Given this context, the aim of the present study was to estimate the flock and animal seroprevalence of *Brucella* infection and to identify potential risk factors in small ruminants in the Emirate of Abu Dhabi. In addition, sera samples tested positive for *Brucella* antibodies by the two ELISA tests were further tested for the genus *Brucella* DNA using quantitative polymerase chain reaction (qPCR). Moreover, genus *Brucella* DNA positive sera samples were tested for *B. abortus*, *B. ovis*, and *B. melitensis* using specific primers.

## 2. Materials and Methods

### 2.1. Description of the Study Area

**Figure 1:**
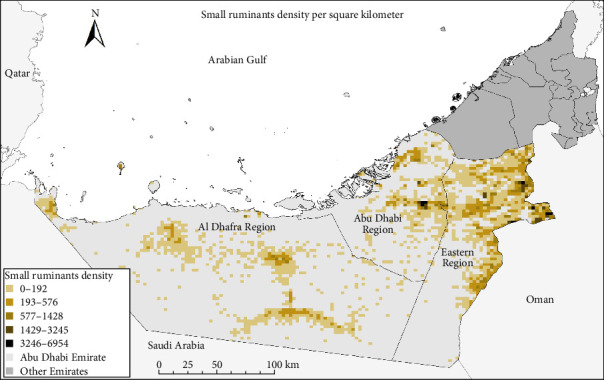
Density of the small ruminant populations in the three regions of the Emirate of Abu Dhabi. The Emirate of Abu Dhabi is subdivided into three regions: the Abu Dhabi, Eastern, and Al Dhafra regions. The density of the small ruminant density of the three regions is variable and it is highest in the eastern regions.

This study was conducted specifically in the Emirate of Abu Dhabi, which has a large geographic area covering 87% of the mainland area of the UAE [16]. The Emirate of Abu Dhabi is divided into three regions: the Abu Dhabi, Eastern, and Al-Dhafra regions (Figure 1). The livestock population in the Emirates has increased sharply in recent years [16]. The total number of sheep and goats in the UAE is estimated to be 5 million [10]. However, the density of small ruminants among the three regions of the Emirate of Abu Dhabi is not uniform. Specifically, the density of small ruminants was relatively higher in the Abu Dhabi and Eastern regions and lower in the Al Dhafra region (Figure 1). In the UAE, the livestock husbandry system is divided into three in the UAE, namely, commercial farming, regular izba and random izba. The regular and random izba types are licensed and have holding numbers; however, they do not have commercial practices (milk or meat). In contrast, farms have commercial practices. Thus, small ruminants are raised on farms for commercial purposes or in izbas for home use. “izba” is a practice that encompasses the traditional way of keeping livestock for personal use at home.

The climate of the UAE is arid, with very high summer (June-September) temperatures and relative humidity levels, reaching 46°C and 100%, respectively [16]. Winter temperatures (December to March) range between 14 and 23°C. The mean annual rainfall is about 78 mm, with over 80% of the annual rain occurring during winter [16].

### 2.2. Sample Size Estimation, Sampling Method, and Distribution of the Samples Across the Three Regions

The sample size calculation was based on the number of individual animals, considering a 2% margin of error and 50% expected seroprevalence. Accordingly, the calculated minimum sample size was 2044; finally, 2730 animals (1298 goats and 1432 sheep) recruited from 272 flocks were sampled, assuming random sampling. Proportional allocation was performed to sample the study animals from flocks. Veterinary clinics found in the three regions were used as sample collection foci. In total, four, eight, and 10 clinics (hospitals) were found in the Abu Dhabi, Al Dhafra, and Eastern regions, respectively. Thus, veterinarians working in clinics traveled to the farms and izbas, located in the surrounding areas to collect the samples.

Body condition scoring (BCS) is used to evaluate the physical condition of an animal based upon muscle and external fat cover. It helps to make management decisions regarding the health of animals and the quality and quantity of feed needed to optimize performance. The body condition of each small ruminant was scored during sample collection, and it was broadly scored as poor, moderate, and good based on the BCS protocols previously published by other researchers [17]. Based on the criteria set by these researchers, the fat and muscle mass of the lumbar, ribs, and sternum areas were considered for the scoring of the body condition of the small ruminants.

### 2.3. Detection of Anti-Brucella Antibodies in the Sera of Small Ruminant Using Competitive Enzyme-Linked Immunosorbent Assay (cELISA) and iELISA Kits

In both ELISA kits, lipopolysaccharide (LPS) antigen pre-coated 96 well plates are used to detect *Brucella* antibodies in the sera. Briefly, cELISA has been designed to detect very low titers of antibodies specific for LPS of *Brucella* species in sera samples of different hosts. Similarly, the iELISA has been designed to detect antibodies against *Brucella* species in hosts. In both kits, the 96 plate wells have been coated with purified *B. abortus* LPS, which is common antigen for all *Brucella* species.

Blood samples (10 mL) were collected from the jugular vein into plain Vacutainers, and stored overnight at room temperature for serum separation. Sera samples were removed from the clotted blood, added into cryovials, labeled, and stored at −80°C until they could be screened for antibodies against *Brucella*. Ingezim *Brucella* Compac 2.0 multispecies (cELISA) and ID Vet multispecies iELISA were applied to detect antibodies in the sera. Animals were classified as young or adult, as their precise ages could not be reliably obtained. The sensitivity and specificity of cELISA were 98.4% (95% CI: 97.0, 99.8) and 99.7% (95% CI: 99.13, 99.61%), respectively [18], while the corresponding values for iELISA were 95.7% (95% CI: 93.4, 98.0) and 99.82% (95% CI: 99.70, 99.95%), respectively [18].

For cELISA, the procedure described by the manufacturer (Ingezim *Brucella* Compac 2.0 multispecies ELISA, Prod. Ref: 10.BRU.K3, Spain) was followed for assay performance. After the assay was completed, the optical density (OD) was measured at 450 nm using a plate reader (BioTek Instrument Inc., Highland Park, USA). The results were validated and interpreted according to the manufacturer's recommendations.

iELISA was conducted following the procedure described by the manufacturer (Innovative Diagnostics, France). After each assay, the OD values were measured at 450 nm (BioTek Instrument Inc., Highland Park, USA). The assay results were validated and interpreted according to the manufacturer's instructions.

### 2.4. Real-Time PCR to Detect Brucella Genus and Species in Seropositive Animals

DNA samples were extracted from the sera of the 39 small ruminants that tested positive for *Brucella* antibodies by both cELISA and iELISA. The NucleoSpin Tissue kit (MACHEREY-NAGEL GmbH & Co.KG, Germany, www.mn-net.com) was used for DNA extraction following the manufacturer's instructions. *Brucella* genus- and species-specific qPCR were performed using the CFX96 Real-Time PCR system (Bio-Rad, Hercules, CA, USA) with SYBR Green chemistry to quantify the amplification of target genes. The target gene for the genus specific qPCR was *Brucella* cell surface 31 kDa protein (*bcsp31*) [19, 20]. The primers used for the amplification of *bcsp31* were forward 5′-GCTCGGTTGCCAATATCAATGC-3′ and reverse-5′-GGGTAAAGCGTCGCCAGAAG-3′ [20, 21]. Samples that were positive for the *Brucella* genus were further tested for three species, namely *Brucella* (*B*). *abortus*, *B. melitensis and B. ovis*. Thee different qPCRs were done for each of the three species separately. The target genes for *B. abortus* and *B. melitensis* qPCRs were *alkB* and *BMEI1162* genes, respectively, both of which are the downstream genes of insertion sequence (IS) 711 (IS711) [20]. The sequences of the primers used for the amplification of these target genes were published earlier [20, 21]. The target gene for *B. ovis* qPCR was *aroA* and the sequences of the primers for its amplification were published by Edao et al. [22]. In addition, glyceraldehyde 3-phosphate dehydrogenase (GAPDH) was amplified using DNA templates as internal control [21]. The primers used for the amplification of GAPDH were: forward-5′-CCACCCATGGCAAATTCC-3′ and reverse-5′-TCGCTCCTGGAAGATGGTG-3′. Instead of probes, Hot Firepolevagreen qPCR Supermix 5x ready-to-use solution (www.solisbiodyne.com) was used to detect the amplified product.

In the genus-specific qPCR, a reaction volume of 12 μl comprises 0.4 μl (10 μM) of each primer, 4 μl (1x) of Hot Firepolevagreen qPCR Supermix, 3 μl of DNA template or GAPDH, and 4.2 μl PCR grade water. The qPCR conditions were as follows: the reaction mixture was first heated at 95°C for 10 min and then subjected to 44 cycles denaturation at 95°C for 15 s and annealing at 60°C for 30 s.

For the species-specific qPCR, reactions were prepared using Maxima SYBR Green qPCR Master Mix (2×; Thermo Fisher Scientific, Waltham, MA, USA). The total volume of the reaction mixture was 25 µL and consisted of 12.5 µL of master mix, 0.3 µM of each forward and reverse primer, 4 µL of template DNA, and nuclease-free water to reach the final volume. The thermal cycling conditions included an initial incubation at 50°C for 2 min, followed by denaturation at 95°C for 10 min. Amplification was carried out over 40 cycles of denaturation at 95°C for 1 s and annealing/extension at 60°C for 30 s.

In both cases, a CFX-96 real-time system (Bio-Rad, Hercules, CA, USA) was used for amplification. The results were captured using the FAM channel, whereas the internal control was read using the HEX channel. The result was considered positive when the quantification cycle (Cq) that is cycle threshold (Ct) value was ≤ 40 [21, 22].

### 2.5. Flowchart of Study Methodology for the Investigation of Brucella Infection in Small Ruminants in the Emirate of Abu Dhabi

**Figure 2:**
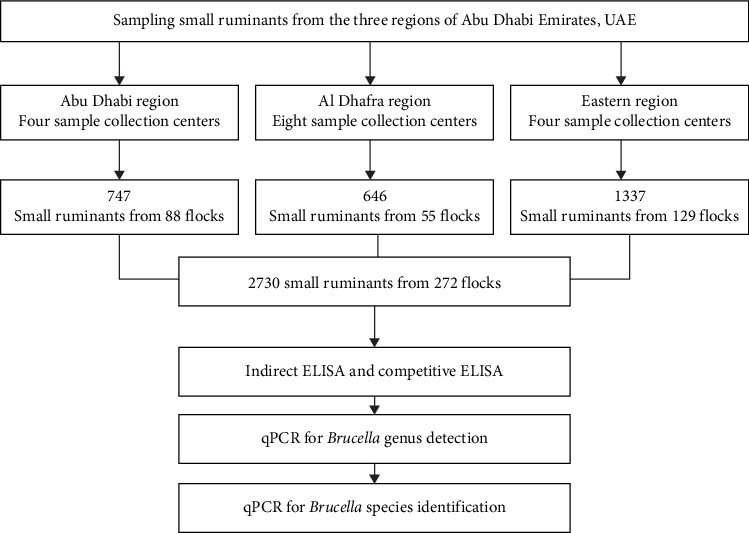
Flowchart of study methodology for the investigation of *Brucella* infection in small ruminants in the Emirate of Abu Dhabi.

The flowchart of the study methodology is presented in Figure 2. The study methodology involved identification of study regions in the Emirate of Abu Dhabi. A total of 22 veterinary clinics were used as focal points to collect 2730 blood samples from the farms and izbas situated in their geographic coverage of animal health care. Sera samples separated and tested for anti-*Brucella* antibodies using both cELISA and iELISA. Sera which were positive by both ELISA tests were tested by qPCR for the genus *Brucella*. *Brucella* genus sera were tested for *B. abortus* and *B. melitensis* using specific primers.

### 2.6. Data Analysis

Animal-level biodata, including species, age, body condition, reproductive status, history of abortion, and origin, were collected during blood sample collection. Serological and qPCR data were obtained from laboratory investigations. SPSS version 21 was used for data analysis. The seroprevalence results are summarized as percentages with 95% confidence intervals. Associations between seroprevalence and potential risk factors were evaluated using binary logistic regression analysis. Variables were selected for multivariate binary logistic regression analysis based on the *p*-value of the univariate logistic regression analysis. Accordingly, variables with *p*-values less than 0.20 were considered in the multivariable binary logistic regression analysis [23]. In all cases, statistical significance was defined by the 95% confidence level with a *p*-value less 0.05.

## 3. Results

### 3.1. Agreement of cELISA and iELISA in Detecting Brucella Infections in Small Ruminants

The test agreement between the cELISA and iELISA in detecting *Brucella* infection in small ruminants is presented in Table 1. The agreement between the two ELISA tests was statistically significant (Kappa = 0.38 ± 0.05; *p* < 0.001). The sensitivities and specificities of the ELISA tests could not be estimated in this study as no gold standard test was used. Nonetheless, cELISA is more sensitive (*χ*^2^ = 25.34; *p* < 0.0001) than iELISA. The higher sensitivity of cELISA leads to the recording of higher flock and animal seroprevalences by cELISA.

### 3.2. Seroprevalence of Brucella Infection and Associated Risk Factors in Flocks of Small Ruminants in the Emirate of Abu Dhabi

Tables 2 and 3 show the seroprevalence of *Brucella* infection in flocks of small ruminants, based on iELISA and cELISA, respectively. Based on iELISA, the seroprevalence of *Brucella* infection in flocks of small ruminants was 13.6% (95% CI: 9.8%–18.3%), while cELISA yielded a seroprevalence of 25.5% (95% CI: 20.3%–31.0%).

Tables 4 and 5 show the associations between the seroprevalence of *Brucella* infection and potential risk factors at the flock level based on iELISA and cELISA, respectively. Overall, iELISA based multivariable binary logistic regression analysis indicated that the odds of *Brucella* infection in medium and large flocks were 3.53 (95% CI: 1.21–10.32) and 4.49 (95% CI: 1.58–12.80) times higher than in small flocks, respectively (Table 4). Similarly, cELISA-based multivariable binary logistic regression analysis revealed that the odds of *Brucella* infection in medium and large flocks were 3.96 (95% CI: 1.72%–9.11%) and 5.24 (95% CI: 2.29–12.0) times higher than in small flocks, respectively (Table 5). Additionally, the odds of *Brucella* infection in the Eastern and Al Dhafra regions were 4.95 (95% CI: 2.11%–11.59%) and 4.04 (95% CI: 1.49%–10.95%) times higher than in the Abu Dhabi region, respectively.

### 3.3. Seroprevalence of Brucella Infection and Associated Risk Factors in Individual Small Ruminants in the Emirate of Abu Dhabi

#### 3.3.1. Seroprevalence Analysis Using Indirect ELISA

The seroprevalence of *Brucella* infection in individual small ruminants was 2.31% (95% CI: 1.8–2.9) based on iELISA (Table 6). Indirect ELISA-based multivariable binary logistic regression analysis revealed that the odds of *Brucella* infection in small ruminants maintained in the Abu Dhabi and Eastern regions were 2.60 (95% CI: 1.01–6.70) and 3.08 (95% CI: 1.26 −7.52) times higher than in small ruminants maintained in the Al Dhafra region, respectively (Table 7). Additionally, the odds of *Brucella* infection in small ruminants kept in farms and regular izbas were 2.62 (95% CI: 1.31–5.26) and 2.60 (95% CI: 1.37–4.91) higher than in small ruminants in random izbas.

#### 3.3.2. Seroprevalence Analysis Using Competitive ELISA

The seroprevalence of *Brucella* infection in small ruminant in the Emirate of Abu Dhabi was 4.84% (95% CI: 4.1–5.7) based on cELISA (Table 8). Multivariable binary logistic regression analysis revealed that the odds of seroprevalence of *Brucella* infection in small ruminants kept in the Eastern region were 2.35 (95% CI: 1.36–4.06) times higher than in small ruminants kept in the Al Dhafra region (Table 9). Furthermore, the odds of the seroprevalence of *Brucella* infection in small ruminants kept in the farm and regular izba were 1.70 (95% CI: 1.07–2.70) and 1.58 (95% CI: 1.04–2.39) times higher than in small ruminants kept in random izbas.

### 3.4. Seroprevalence of Brucella Infection in Small Ruminants at the Clinic (hospital) Level in the Emirate of Abu Dhabi

**Figure 3:**
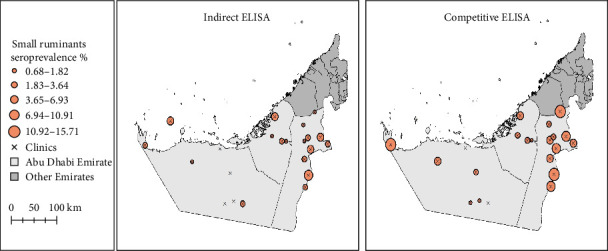
Seroprevalences of *Brucella* infection in small ruminants in the areas surrounding veterinary clinics (hospitals) of the three regions in the Emirate of Abu Dhabi. The seroprevalence values at surrounding the different clinics are indicated by circles with different sizes. while the clinics are indicated with an x mark. The sizes of the circles indicate the magnitude of the seroprevalence values; the larger the circle the higher the seroprevalence.

The seroprevalence of *Brucella* infection in small ruminants in animal holdings located in the clinic (hospital) across the three target regions is presented in Figure 3. Overall, there were differences in seroprevalence among holdings located in different clinics (hospitals). In the Abu Dhabi region, the highest seroprevalence was recorded in animal holdings located around Samha Veterinary Clinic, whereas the lowest was recorded in animal holdings located around Khazna Veterinary Clinic. In the eastern region, the highest seroprevalence was recorded in the animal holdings surrounding the Wagan Veterinary Clinic, while the lowest was recorded in those surrounding the Abu Samra, Araad, and Sösabra Veterinary Clinics. In contrast, the seroprevalence was low in animal holdings located in most veterinary clinics in the Al-Dhafra region. In this region, a relatively high seroprevalence was recorded in animal holdings surrounding the Selaa Veterinary Clinic.

### 3.5. Detection of Brucella Genus and Species Using qPCR in the Sera of Seropositive Small Ruminants

Quantitative PCR detected the *Brucella* genus in 28.2% (11/39) of the seropositive small ruminants (Table 10). Nine of the 11 DNA-positive serum samples were from sheep, whereas the remaining two were from goats. The *Brucella* genus positive sera were tested for *Brucella* species using specific primers of three species (*B. abortus*, *B. melitensis*, and *B. ovis*). *B. melitensis* was detected in five sera samples (in two as a single infection and three mixed infections). The second common species was *B. ovis* that was detected in four sera samples (in three as a single infection and in one mixed infection). Lastly, *B*. *abortus* was detected in three sera samples (in one as a single infection and in two as mixed infections with *B. melitensis*. Two *Brucella* genus positive sera were not positive for any of these three species of *Brucella*. The mean ± standard error of mean (SEM) of Cq of positive *Brucella* genus qPCR was 38.44 ± 0.33 (95% CI: 37.7–39.2) while that of the internal control (GAPDH) was 32.38 ± 0.36 (95% CI: 31.49–33.27). The means of Cq were not calculated for the qPCR of each species, as the number of each species was low.

## 4. Discussion

In this article, we present the results of an epidemiological study conducted to assess the situation of *Brucella* infection using serological tests in 272 flocks comprising 2730 small ruminants in three regions of the Emirate of Abu Dhabi between 2022 and 2023. In addition, sera samples positive by the ELISA tests were further tested for *Brucella* DNA using qPCR. The result of this study and earlier similar study recently published by our team [24] could serve as a basis for future studies on the situation of *Brucella* infection in the Emirate of Abu Dhabi, as both studies were comprehensive and conducted on large sample sizes and covered all the three regions of the Emirate of Abu Dhabi of the UAE.

Based on the iELISA results, the flock seroprevalence was 13.6%; while it was 25.5% on the basis of cELISA. The difference in seroprevalences recorded by the ELISA tests was due to the difference in the sensitivities of the two ELISA tests. The higher seroprevalence recorded by cELISA could due its higher sensitivity. The sensitivity and specificity of cELISA were 98.4% and 99.7%, respectively, while the corresponding values for iELISA were 95.7% and 99.82%, respectively [18]. Similar studies were conducted in the flocks of ruminants elsewhere. Studies conducted in Jordan, Spain, Algeria, and Egypt reported similar seroprevalence values [25–28]. On the other hand, higher seroprevalence values were reported from Eretria, Tunisia, and Palestine [13, 29]. In general, the difference in the seroprevalence of *Brucella* infection in flocks from different countries could be due to the difference in the sensitivities of tests used, the flock size and the difference between countries in implementing control and preventive measures.

Individual animal seroprevalence was 2.31% based on iELISA and 4.84% based on cELISA. Thus, relatively higher individual animal seroprevalence was recorded by cELISA than iELISA, which is mainly due to the higher sensitivity of cELISA as indicated above. Thus, similar to the difference in seroprevalence recorded by the two ELISA types, the type of ELISA used for the different studies affects the seroprevalence reports. A few studies have reported animal seroprevalence of *Brucella* infection in small ruminants in the UAE [11–14]. Refai [13] reported animal seroprevalences of 6.4% and 5.4% in goats and sheep, respectively, in 1989 in the UAE. Furthermore, the same author reported animal seroprevalences of 3.4% and 2.0% in goats and sheep, respectively, in 1990 [13]. Conversely, a higher (8.4%) animal seroprevalence was reported in small ruminants from the UAE [14].

In addition to the studies reported from the UAE, several studies reported various levels of seroprevalence of *Brucella* infection in small ruminants from countries in the Middle East, East Africa, North Africa, and Southeast Asia [30–37]. According to a review published in Pakistan [38], the seroprevalence of *Brucella* infection in small ruminants varies from 7.1% to 35.1%, depending on the type of diagnostic test, the farming system, and various environmental factors. Another review article published on *Brucella* infection in small ruminants in Iran reported animal seroprevalences of 4% and 5% in sheep and goats, respectively [39]. In Jordan, one sero-surveillance study of brucellosis in 1100 goats recorded a seroprevalence of 27.7%, which could be classified as a high seroprevalence [40].

In general, the seroprevalence values recorded by this study at the animal level by both ELISA tests were less than 5% and thus could be considered low as compared to the seroprevalence values reported from the other countries in the Middle East, which is possibly due to the implementation of the test-and-slaughter strategy in the UAE [41]. The UAE's National Animal Health Plan for the Control of *Brucella* infection includes the eradication of the infection from the country through adaptation of the test and slaughter policy. However, a review published on the control of *Brucella* infection earlier [42] indicated that the test-and-slaughter strategy has not been successful in some Middle East countries as it was not complemented with vaccination.

Animal-level seroprevalence varied among the different regions of the Abu Dhabi Emirate. The lowest seroprevalence was recorded in the Al Dhafra region, and a similar observation was made earlier in a similar study [14]. The low seroprevalence in the AlDhafra region could be due to the low density of small ruminant population in the Al Dhafra region as compared the small ruminant density in the Eastern and Abu Dhabi regions. In addition, the Al Dhafra region is characterized by a desert ecology, which could limit the transmission of *Brucella* infection. In addition, differences in the seroprevalence of *Brucella* infections were observed at clinics (hospitals) that served as focal sites for sample collection. This variation in the seroprevalence of *Brucella* infection in clinics (hospitals) within each region could also be associated with differences in the densities of animal populations.

The genus *Brucella* was detected in 11 of 39 seropositive small ruminants. *B. melitensis* and *B. abortus* were isolated from the seropositive animals. Different authors have previously detected *Brucella* DNA in the sera of seropositive animals [21, 43–47]. In one Italian study investigating 53 seropositive animals, 35 (66%) tested positive for *Brucella* DNA [43]. In contrast, all 25 serum samples from 25 aborted livestock species were positive for *Brucella* DNA, which could be due to an ongoing active infection [44]. One study indicated that the sensitivity of qPCR was 70.2% in positive sera, but 97.2% in culture positive blood [21]. Conversely, studies from Egypt [45] and Pakistan [46] reported that qPCR *Brucella* DNA was identified in 27% (32/118) and 44% (31/71) of seropositive animals, respectively. As can be seen from the results of these studies, the proportions of the detection of *Brucella* DNA in the sera of seropositive animals are variable. This difference may be predominantly associated with the stage of *Brucella* infection. In the case of active infection and bacteremia, the chance of detecting *Brucella* DNA in the serum is higher, whereas in latent infection, where the infection is localized to a specific lymph node, the chance of detecting bacterial DNA in the serum is less likely.

In the present study, the DNA of *B. melitensis*, *B. ovis*, and *B. abortus* was detected in the sera samples. Mixed infection with *B. melitensis* and either *B. ovis* or *B. abortus* were also observed in three small ruminant sera. Overall, *B. melitensis* was the dominant species detected followed with *B. ovis*. The detection of *B. melitensis* is significant not only because its economic impact in small ruminant production but also because of the severe disease that it causes in humans. Similarly, earlier studies have also reported the isolation of *B. meltensis* from sheep and sheep farm workers in the Al Ain district of the UAE [11, 12]. *B. melitensis* has more frequently been isolated from goats and ewes, while *B. ovis* is common in rams [48]. *B. melitensis* causes abortion, stillbirth, weak offspring, and epididymal orchitis in goats and sheep [49]. In contrast, *B. ovis* primarily infects rams, causing epididymitis, but rarely causes abortions in ewes [50]. *B. ovis* infection is transient in both male and female goats [51]. Regarding zoonosis, *B. melitensis* is the most virulent *Brucella* species in humans and is responsible for a severely debilitating and disabling illness that results in high morbidity and relatively low mortality, whereas *B. ovis* is nonpathogenic in humans [52].

Besides livestock species, several prior studies have reported on the occurrence of *Brucella* infections in wild and captive animals in the UAE [41, 53, 54]. *B. melitensis* has been isolated from the swollen metacarpal joint of a reem gazelle and a severe orchitis lesion of an Oryx antelope [41]. More recently, a seroprevalence of 67% for *Brucella* infection was reported in one study investigating 959 captive scimitar-horned oryxes in the country, while *B. melitensis* biovar 1 was isolated from a captive wildlife population [53, 54]. Such isolation of *Brucella* species from wild and captive animals underscores their significance as potential sources of *Brucella* infection in both domestic animals and humans.

On top of its significance in livestock and wildlife, *Brucella* infection has also been reported in humans in the UAE [11, 12, 55–57]. Three decades ago, an outbreak of human brucellosis was recorded in Al-Ain [11]. During this outbreak, *B. melitensis* biovar 2 was first isolated from a 45-year-old woman in the UAE in 1996 [12]. Additionally, 480 human cases of brucellosis were reported in one retrospective that covered 15 years (2000–2015) [55]. A similar retrospective study reported 91 pediatric cases of brucellosis at Tawam Hospital between 2009 and 2017 [56]. The authors indicated that most pediatric patients consumed unpasteurized camel's milk. Furthermore, neurobrucellosis was diagnosed in Ras Al-Khaimah in the UAE in a patient who had a history of ingesting unpasteurized camel milk [57]. Another retrospective study reported 99 cases of human brucellosis at Tawam Hospital between 2009 and 2016 [58]. The results of both the present and previous studies indicate that brucellosis is an important endemic disease in the UAE, where it may have significant effects on public and animal health.

## 5. Conclusion

Overall, the flock seroprevalence of *Brucella* infection identified in the present study was considered moderate, whereas the animal seroprevalence was considered low. Flock seroprevalence was associated with flock size, whereas animal seroprevalence was associated with region, type of animal holding, and animal species and age. Besides, *B. melitensis*, *B. ovis*, and *B. abortus* were detected in seropositive small ruminants, which could suggest the potential threat of these pathogens to the public health and livestock production. Thus, the results of this study could suggest the need or enforce preventative and control measures of *Brucella* infection to minimize the economic and public health impacts of *Brucella* infection. Test-and-slaughter and vaccination could be considered as sound control and preventive measures.

## Figures and Tables

**Table 1:** Test agreement between cELISA and iELISA in detecting *Brucella* infections in small ruminants in the United Arab Emirates.

	iELISA		Total	Weighted Kappa ± SE	95% confidence interval	*Z* test	*p*-Value
	Negative	Positive					
cELISA							
Negative	2574	24	2598	0.38 ± 0.05	0.293–0.469	21.37	<0.001
Positive	93	39	132	—	—	—	—
Total	2667	63	2730	—	—	—	—

Animal prevalence by cELISA		4.84%		—	—	—	—
Animal prevalence by iELISA		2.31%		—	—	—	—
Difference in prevalence		2.53%		—	—	—	—
*χ* ^2^		25.34		—	—	—	—
*p* Value		<0.0001		—	—	—	—

**Table 2:** Indirect ELISA-based seroprevalence of *Brucella* infection in flocks of small ruminants in the Emirate of Abu Dhabi.

Risk factor	Category	No. of tested flock	No of positive flock	Percent positive (95% CI)	*χ* ^2^	*p* Value
Region	Abu Dhabi	88	9	10.2 (4.8 −18.5)	2.788	0.225
	Eastern	129	17	13.2 (7.9–20.3)		
	Al Dhafra	55	11	20.0 (10.4–33.0)		

Holding type	Farm	55	5	9.1 (3.0–20.0)	3.077	0.215
	Regular izba	126	22	17.5 (11.3–25.2)		
	Random izba	91	10	11.0 (5.4–19.3)		

Herd size	Small (≤ 20)	97	5	5.2 (1.7–11.6)	10.162	0.006
	Medium (20–50)	89	14	15.7 (8.9–25.0)		
	Large (≥ 50)	86	18	20.9 (12.9–31.0)		

Mixed with camels	No	177	24	13.6 (8.9–19.5)	0.001	1
	Yes	95	13	13.7 (7.5–22.3)		

Total		272	37	13.6 (9.8–18.3)	—	—

**Table 3:** Competitive ELISA-based seroprevalence of *Brucella* infection in flocks of small ruminants in the Emirate of Abu Dhabi.

Risk factor	Category	No. of the tested flocks	No of positive flocks	Percent of positive (95% CI)	*χ* ^2^	*p* Value
Region	Abu Dhabi	88	9	10.2 (4.8 −18.5)	18.412	<0.0001
	Eastern	129	44	34.1 (26.0–43.0)		
	Al Dhafra	55	17	30.9 (19.1–44.8)		

Holding type	Farm	55	8	14.5 (6.5–26.7)	8.594	0.013
	Regular izba	126	42	33.3 (25.2–42.3)		
	Random izba	91	19	20.9 (13.1–30.7)		

Herd size	Small (≤ 20)	97	10	10.3 (5.1–18.1)	21.083	<0.0001
	Medium (20–50)	89	25	28.1 (19.1–38.6)		
	Large (≥ 50)	86	34	39.5 (29.2–50.7)		

Mixed with camels	No	177	38	21.5 (15.7–28.3)	4.069	0.057
	Yes	95	31	32.6 (23.4 −43.0)		

Total		272	69	25.4 (20.3–31.0)	—	—

**Table 4:** Indirect ELISA-based binary logistic regression analysis of the association of potential risk factors with the seroprevalence of *Brucella* infection in the flocks of small ruminants.

Risk factor	Category	No. of tested flock	No of positive flocks	Crude OR		Adjusted OR	
				OR (95% CI)	*p* Value	OR (95% CI)	*p* Value
Region	Abu Dhabi	88	9	1	—	1	—
	Eastern	129	17	1.33 (0.57–3.14)	0.512	1.16 (0.47–2.85)	0.748
	Al Dhafra	55	11	2.19 (0.84–5.70)	0.107	1.81 (0.64–5.07)	0.262

Holding type	Farm	55	5	1	—	1	—
	Regular izba	126	22	2.12 (0.76–5.91)	0.153	1.85 (0.64–5.36)	0.255
	Random izba	91	10	1.24 (0.40–3.81)	0.715	1.26 (0.39–4.05)	0.68

Herd size	Small (≤ 20)	97	5	1	—	1	—
	Medium (20–50)	89	14	3.44 (1.18–9.97)	0.023	3.53 (1.21–10.32)	0.021
	Large (≥ 50)	86	18	4.87 (1.72, 13.77)	0.003	4.49 (1.58–12.80)	0.005

**Table 5:** Competitive ELISA-based binary logistic regression analysis of the association of potential risk factor with the seroprevalence of *Brucella* infection in the flocks of small ruminants.

Risk factor	Category	No. of tested flocks	No of positive flocks	Crude OR		Adjusted OR	
				OR (95% CI)	*p* Value	OR (95% CI)	*p* Value
Region	Abu Dhabi	88	9	1	—	1	—
	Eastern	129	44	5.18 (2.30–11.67)	<0.001	4.95 (2.11–11.59)	<0.001
	Al Dhafra	55	17	4.47 (1.77–11.28)	0.001	4.04 (1.49–10.95)	0.006

Holding type	Farm	55	8	1	—	1	—
	Regular izba	126	42	2.94 (1.27–6.78)	0.012	2.15 (0.88–5.27)	0.095
	Random izba	91	19	1.55 (0.63–3.83)	0.342	1.56 (0.58–4.15)	0.375

Herd size	Small (≤ 20)	97	10	1	—	1	—
	Medium (20–50)	89	25	3.40 (1.53–7.57)	0.003	3.96 (1.72–9.11)	0.001
	Large (≥ 50)	86	34	5.69 (2.60–12.46)	<0.0001	5.24 (2.29–12.0)	<0.00

Mixed with camels	No	177	38	1	—	1	—
	Yes	95	31	1.77 (1.01–3.10)	0.045	1.25 (0.66–2.36)	0.489

**Table 6:** Indirect iELISA-based univariable binary logistic regression analysis of the association potential risk factors with the seroprevalence of *Brucella* infection in small ruminants.

Risk factor	ID vet ELISA test		Total	Prevalence (%)	95% CI	Odd ratio (95% CI)	*χ* ^2^	*p* Value
	Negative	Positive						
Region								
Abu Dhabi	725	22	747	2.95	1.9–4.4	3.24 (1.30–8.03)	7.365	0.025
Eastern	1302	35	1337	2.62	1.8–3.6	2.87 (1.20–6.85)	—	—
Al Dhafra	640	6	646	0.93	0.3–2.0	1	—	—
Total	2667	63	2730	2.31	1.8–2.9	—	—	—
Type of holding								
Farm	633	21	654	3.21	2.0–4.9	2.70 (1.40–5.21)	13.596	0.001
Regular izba	732	26	758	3.43	2.3–5.0	2.89 (1.54–5.42)	—	—
Random izba	1302	16	1318	1.21	0.7–2.0	1	—	—
Species								
Sheep	1391	41	1432	2.86	2.1–3.9	1.71 (1.01–2.89)	4.122	0.055
Goat	1276	22	1298	1.69	1.1–2.6	1	—	—
Age								
Young	685	17	702	2.42	1.4–3.9	1	0.054	0.884
Adult	1982	46	2028	2.27	1.7–3.0	1.08 (0.15–8.04)	—	—
Body condition								
Good	55	1	56	1.79	0.0–9.6	1	4.709^a^	0.091
Moderate	684	24	708	3.39	2.2–5.0	1.93 (0.26–14.54)	—	—
Poor	1928	38	1966	1.93	1.4–2.6	1.08 (0.15–8.04)	—	—
Status of reproduction								
Dry	1047	21	1068	1.97	1.2–3.0	1	3.163^a^	0.366
Pregnant	599	20	619	3.23	2.0, 5.0	1.67 (0.90–3.10)	—	—
Lactating	823	17	840	2.02	1.2–3.2	1.03 (0.54–1.97)	—	—
Young	198	5	203	2.46	0.8–5.7	1.26 (0.47–3.38)	—	—
History of abortion								
Yes	21	0	21	—	0.0–16.1	0	^a^	> 0.999
No	2484	62	2546	2.50	1.9–3.1	1	—	—
Origin of animal								
Born in	2592	63	2655	2.43	1.8–3.0	1	^a^	0.418
Introduced	75	0	75	—	0.0–4.8	0	—	—

^a^Fischer exact test.

**Table 7:** Results of iELISA-based multivariable binary logistic regression analysis of the association of potential risk factors with the seroprevalence of *Brucella* infection in small ruminants.

Risk factor	Category	Odd ratio (95% CI)	Wald test	df	*p* Value
Region		—	6.123	2	0.047
	Abu Dhabi	2.60 (1.01–6.70)	3.935	1	0.047
	Eastern	3.08 (1.26, 7.52)	6.123	1	0.013
	Al Dhafra	1	—	—	—

Type of holding		—	10.069	2	0.007
	Farm	2.62 (1.31–5.26)	7.346	1	0.007
	Regular izba	2.60 (1.37–4.91)	8.562	1	0.003
	Random izba	1	—	—	—

Species	Sheep	—	—	—	—
	Goats	1.66 (0.98–2,82)	3.521	—	0.061

Body condition		—	7.51	2	0.023
	Good	1	0.293	1	0.589
	Moderate	1.76 (0.23–13.74)	0.028	1	8.68
	Poor	0.84 (0.11–6.49)	—	—	—

**Table 8:** Results of cELISA-based univariable binary logistic regression analysis of the association of potential risk factor with the seroprevalence of *Brucella* infection in small ruminants.

Risk factor	Competitive ELISA		Total	Prevalence (%)	95% CI	Odd ratio (95% CI)	*χ* ^2^	*p* Value
	Negative	Positive						
Region								
Abu Dhabi	722	25	747	3.35	2.2–4.9	1.28 (0.69–2.394)	20.863	<0.0001
Eastern	1247	90	1337	6.73	5.4–8.2	2.67 (1.58–4.52)	—	—
Al Dhafra	629	17	646	2.63	1.5–4.2	1	—	—
Total	2598	132	2730	4.84	4.1–5.7	—	—	—
Type of holding								
Farm	620	34	654	5.20	3.6–7.2	1.34 (0.86–2.08)	4.962	0.084
Regular izba	712	46	758	6.07	4.5–8.0	1,57 (1.05–2.36)	—	—
Random izba	1266	52	1318	3.95	3.0–5.1	1	—	—
Species								
Sheep	1362	70	1432	4.89	3.8–6.1	1.03 (0.72–1.46)	0.018	0.929
Goat	1236	62	1298	4.78	3.7–6.1	1	—	—
Age								
Young	680	22	702	3.13	2.0–4.7	1	5.944	0.018
Adult	1918	110	2028	5.42	4.5–6.5	1.77 (1.11–2.83)	—	—
Body condition								
Good	54	2	56	3.57	0.4–12.3	1	1.364^a^	0.473
Moderate	668	40	708	5.65	4.1–7.6	1.62 (0.38–6.87)	—	—
Poor	1876	90	1966	4.58	3.7–5.6	1.30 (0.31–5.40)	—	—
Status of reproduction								
Dry	1020	48	1068	4.49	3.3–5.9	1	1.744	0.628
Pregnant	583	36	619	5.82	4.1–8.0	1.31 (0.84–2.05)	—	—
Lactating	802	38	840	4.52	3.2–6.2	1.01 (0.65–1.56)	—	—
Young	193	10	203	4.93	2.4–8.9	1.10 (0.55–2.21)	—	—
History of abortion								
Yes	20	1	21	4.76	0.1–23.8	1	^a^	> 0.999
No	2420	126	2546	4.95	4.1–5.9	1.04 (0.14–7.82)	—	—
Origin of animal								
Born in	2524	131	2655	4.93	4.1–5.8	3.84 (0.53–27.84)	^a^	0.265
Introduced	74	1	75	1.33	0.0–7.2	1	—	—

^a^Fischer exact test.

**Table 9:** Results of cELISA-based multivariable binary logistic regression analysis of the association of potential risk factors with the seroprevalence of *Brucella* infection in small ruminants.

Risk factor	Category	Odd ratio (95% CI)	Wald test	df	*p* Value
Region		—	17.501	2	<0.0001
	Abu Dhabi	1.01 (0.53–1.92)	0.001	1	0.975
	Eastern	2.35 (1.36–4.06)	9.282	1	0.002
	Al Dhafra	1	—	—	—

Type of holding		—	6.637	2	0.036
	Farm	1.70 (1.07–2.70)	5.006	1	0.025
	Regular izba	1.58 (1.04–2.39)	4.558	1	0.033
	Random izba	1	—	—	—

Age	Young	—	—	—	—
	Adult	1.37 (0.84–2.22)	1.563	1	0.211

**Table 10:** *Brucella* species detected in the seropositive small ruminants in the Emirate of Abu Dhabi in the UAE.

Lab ID	Regions	Animal species	cELISA	iELISA	Genus qPCR	Ct value (genus)	Species qPCR	Ct value (species)
68	Eastern	Goat	Positive	Positive	Positive	36.8	*B. melitensis*	38.74
360	Eastern	Sheep	Positive	Positive	Positive	38.25	Negative	Negative
362	Eastern	Sheep	Positive	Positive	Positive	39.41	*B. abortus* and *B. melitensis*	40.0 and 37.1
363	Eastern	Sheep	Positive	Positive	Positive	38.89	*B. ovis*	38.78
591	Eastern	Sheep	Positive	Positive	Positive	39.58	*B. melitensis*	40.0
757	Eastern	Goat	Positive	Positive	Positive	38.2	*B. abortus*	35.7
1366	Abu Dhabi	Sheep	Positive	Positive	Positive	39.44	*B. abortus* and *B. melitensis*	39.1 and 38.7
1373	Abu Dhabi	Sheep	Positive	Positive	Positive	37.45	Negative	Negative
1374	Abu Dhabi	Sheep	Positive	Positive	Positive	37.42	*B. ovis*	36.97
2362	Abu Dhabi	Sheep	Positive	Positive	Positive	37.42	*B. melitensis* and *B. ovis*	40.0 and 40,0
2364	Abu Dhabi	Sheep	Positive	Positive	Positive	40.00	*B. ovis*	—
GAPDH	Positive control	—	—	—	—	—	—	33.71
GAPDH	Positive control	—	—	—	—	—	—	32.90
No Template control	Negative control	—	—	—	—	—	—	Negative
No Template control	Negative control	—	—	—	—	—	—	Negative

Abbreviation: GAPDH, glyceraldehyde 3-phosphate dehydrogenase.

## Data Availability

The data that support the findings of this study are available from the corresponding author upon reasonable request.
